# Dietary Restraint: Far Too Complex to Dismiss as a Fallacy

**DOI:** 10.1002/eat.24582

**Published:** 2025-10-23

**Authors:** Marle Alvarenga, César Moraes

**Affiliations:** ^1^ Postgraduate Program in Nutrition in Public Health School of Public Health, University of São Paulo São Paulo Brazil; ^2^ São Francisco University Bragança Paulista Brazil

**Keywords:** calorie restriction, dietary restraint, eating disorders, obesity

## Abstract

In her commentary *Dietary Restraint Fallacy*, Jansen argues that the theory linking dietary restraint to binge eating and weight gain can be dismissed as a “fallacy.” This conclusion is largely drawn from Grilo and Pittman, who found that rigid restraint was associated with reduced binge frequency and greater weight loss. We contend that dietary restraint remains a profoundly complex and unsettled issue, with implications for weight regain and eating disorder prevention. Importantly, the three Commentaries on Grilo and Pittman were invited simultaneously. Readers encountering only one of them may not realize that these papers were designed to present contrasting viewpoints. In this Commentary, we stress why restraint theory cannot be dismissed wholesale, highlight the need for integration between obesity and eating disorders fields, and emphasize that premature simplifications risk distorting both scientific understanding and clinical practice.

## RESUMEN

En su comentario *Dietary Restraint Fallacy*, Jansen sostiene que la teoría que vincula la restricción dietética con los atracones y el aumento de peso puede desestimarse como una “falacia.” Esta conclusión se basa en gran medida en Grilo y Pittman, quienes hallaron que la restricción rígida se asociaba con una menor frecuencia de atracones y una mayor pérdida de peso. Sostenemos que la restricción dietética sigue siendo un tema profundamente complejo y no resuelto, con implicaciones para la recuperación de peso y la prevención de los trastornos alimentarios. Es importante destacar que los tres Comentarios sobre Grilo y Pittman fueron invitados simultáneamente. Los lectores que se encuentren con solo uno de ellos pueden no percibir que estos artículos fueron concebidos para presentar puntos de vista contrastantes. En este Comentario, subrayamos por qué la teoría de la restricción no puede descartarse de manera general, destacamos la necesidad de integrar los campos de la obesidad y los trastornos alimentarios y enfatizamos que las simplificaciones prematuras corren el riesgo de distorsionar tanto la comprensión científica como la práctica clínica.

## 1 Introduction

We recognize the value of critical debate in the study of eating behavior. Yet, both the provocative title and the central premise of Jansen's *Dietary Restraint Fallacy* (Jansen 2025) risk oversimplifying a topic that is inherently complex. For readers unfamiliar with the editorial context, it may not be clear that Jansen's Commentary was one of three simultaneous responses to Grilo and Pittman (2024). Without this information, the label “fallacy” may be mistakenly interpreted as consensus rather than as one perspective among deliberately divergent views. Our goal is to contribute to a constructive integration of perspectives, encouraging dialog between obesity and eating disorder research traditions rather than positioning one against the other.

Restraint has a long history in the literature as a construct implicated in the onset, maintenance, and relapse of disordered eating and weight problems. To frame it as a fallacy risks obscuring this evidence and the clinical realities observed in practice.

## 2 The Complexity of Dietary Restraint

Dietary restraint is far from a unitary phenomenon. It has been defined variously as cognitive restraint, caloric restriction, restrained eating, or the use of rigid versus flexible control strategies. As Mills et al. (2021) argue, dieting and restrained eating are not synonymous, and conflating them obscures both theory and clinical application.

Restrained eating has been recognized as a precipitating factor in disordered eating, though not a sole “cause.” Etiology in eating disorders is multifactorial and graded: factors act as predisposing, precipitating, or perpetuating influences depending on context and timing.

Applying this lens to Grilo and Pittman (2024), several methodological and conceptual issues warrant consideration. First, the *etiologic window* in their study is relatively short; such a brief follow‐up may capture immediate reductions in binge frequency but not the risk of longer‐term relapse. Second, the possibility of *reverse causality* should be acknowledged, early improvements in binge symptoms may enable greater adherence to restraint, rather than restraint producing those improvements. Finally, the distinction between *prevalence and incidence* is crucial: clinical trial samples differ markedly from population‐level patterns, where dieting typically precedes the onset of disordered eating. Taken together, these considerations underscore why restraint theory cannot be reduced to a binary verdict of “fallacy.”

Trait versus state distinctions further illustrate the problem: instruments for “restrained eating” often mix heterogeneous items, obscuring whether findings reflect stable dispositions or transient dieting efforts. Similarly, cumulative dieting history may influence outcomes long after specific diets end. Not all restrictions are pathological; for example, in medical contexts such as diabetes or celiac disease, but the category requires careful contextualization.

## 3 Multiple Perspectives in the Debate

The current debate illustrates the breadth of interpretation around restraint. Jansen (2025) dismissed restraint theory as a fallacy. By contrast, Levinson et al. (2025) provocatively argued that *behavioral weight loss promotes weight loss by increasing eating disorder symptoms*. Balantekin and Hayes (2025) emphasized the distinction between structured behavioral interventions and unsupervised dieting, noting that not all forms of rigid restraint are maladaptive.

These Commentaries, though published in different months, were invited at the same time. Authors did not have access to each other's responses, and the simultaneity may not be apparent to readers. This context matters: without it, an individual Commentary may appear definitive rather than one voice among several divergent perspectives.

## 4 Implications for Weight Regain and Prevention

Even when structured interventions yield short‐term weight loss and reduced binge symptoms, the challenge of weight regain is pervasive. Rigid restraint may contribute to weight cycling, relapse, and persistent dissatisfaction, perpetuating rather than resolving difficulties.

Jansen's conclusion that prevention strategies should promote “healthy calorie restriction” oversimplifies a complex issue. The field lacks consensus on what constitutes “healthy” caloric intake, especially across developmental stages. While the obesity field rightly emphasizes the risks of caloric overconsumption, eating disorders research shows that dieting behaviors are established risk factors. Recommending caloric restriction as a cornerstone of prevention risks obscuring this nuance, particularly among youth populations.

Epidemiological patterns further complicate the picture: despite decades of public health emphasis on calorie restriction, prevalence rates of both obesity and eating disorders have continued to rise. This contradiction suggests that restriction‐focused strategies may be insufficient and, in some cases, counterproductive.

## 5 Conclusion

The debate surrounding restraint theory remains unsettled. Simplistic framings—whether declaring restraint a “fallacy” or arguing it uniformly increases disorder symptoms—do not capture the full complexity of evidence and clinical practice.

Our concern is not that a single Commentary could overturn decades of research, but that provocative rhetoric, when read without awareness of the editorial context, may create misleading impressions. We, therefore, call for careful attention to definition, measurement, and context, as well as integration across obesity and eating disorder fields. Only through this nuance can we advance prevention and treatment strategies that address both metabolic and psychological well‐being.

## Author Contributions

**Marle Alvarenga:** conceptualization, writing – original draft, validation, writing – review and editing. **César Moraes:** conceptualization, writing – original draft, validation, writing – review and editing.

## Data Availability

Data sharing not applicable to this article as no datasets were generated or analysed during the current study.
